# Determinants of frequency and contents of antenatal care visits in Bangladesh: Assessing the extent of compliance with the WHO recommendations

**DOI:** 10.1371/journal.pone.0204752

**Published:** 2018-09-27

**Authors:** M. Mazharul Islam, Mohammad Shahed Masud

**Affiliations:** 1 Department of Statistics, College of Science, Sultan Qaboos University, Muscat, Oman; 2 Institute of Statistical Research and Training (ISRT), University of Dhaka, Dhaka, Bangladesh; Obafemi Awolowo University, NIGERIA

## Abstract

**Background:**

In addition to the number of antenatal care (ANC) visits, the items of ANC services covered by ANC visits greatly influence the effectiveness of the ANC services. Recently the World Health Organization (WHO) recommended not only to achieve a minimum of eight ANC visits, but also to use a core set of items of ANC services for safe motherhood. This study examined the levels and determinants of frequency and contents of ANC visits in Bangladesh and thus assessed the level of compliance with the WHO recommended number and the content of ANC services during pregnancy in Bangladesh.

**Methods:**

The data for the study come from the 2014 Bangladesh Demographic and Health Survey (BDHS), which covereda nationally representative sample of 17,863 ever-married women aged 15–49 years. Data derived from 4,627 mothers who gave birth in the three years preceding the survey constituted the study subjects. Descriptive, inferential and multivariate statistical techniques were used for data analysis.

**Results:**

On average, mothers received less than three (2.7 visits) ANC visits and only 6% receive the recommended eight or more ANC visits. About 22% of the mothers received all the prescribed basic items of ANC services. About one-fifth (21%) of the mothers never received ANC visits and thus no items of ANC services. Measurement of blood pressure was the most common item received during ANC visit as reported by 69% mothers. Blood test was the least received item (43%). Significant positive association was found between frequency of ANC visits and receiving the increased number of items of ANC services. High socio-economic status, low parity, living in urban areas and certain administrative regions, planned pregnancies, having media exposure, visiting skilled providers for ANC services and visit to public or NGO health facilities are associated with frequent ANC visits and receiving higher number of items of ANC contents.

**Conclusion:**

An unsatisfactory level of coverage of and content of ANC visits have been observed in Bangladesh. Further investigation is needed to identify the causes of under-utilization of ANC services in Bangladesh. A greater understanding of the identified risk factors and incorporating them into short and long term strategies would help improve the coverage and contents and thus quality of ANC services in Bangladesh.

## Introduction

All pregnancies are deemed to carry some risks of complications and adverse outcomes—some carry less risk, while others carry more risks[1,2]. Complications during pregnancy, childbirth and puerperium are the leading causes of deaths and disabilities among women of child bearing age [2]. According to a systematic analysis by the United Nation (UN) Maternal Mortality Estimation Inter-Agency Group, in 2015 approximately 830 women die every day globally due to complications during pregnancy or childbirth; approximately 99% of these deaths take place in developing countries [3,4]. On the other hand, approximately 2.6 million pregnancies were terminated as stillbirth in 2015, also mainly in developing countries [5]. However, pregnancy related complications and deaths are not inevitable; most of them are preventable with simple and cost-effective maternity care during pregnancy, delivery and postnatal period [6,7]. Within the continuum of maternity care, antenatal care (ANC) provides a platform for important health-care functions, including health promotion, screening and diagnosis, and disease prevention [8]. Some studies have estimated that ANC alone can reduce maternal mortality by 20%, given good quality and regular attendance[1,9].

The purpose of ANC is to monitor and safeguard the wellbeing of the mother and foetus, detect any pregnancy complications and take necessary measures, respond to mother’s complaints, prepare mother for birth, and promote healthy behaviors of mother[2, 8]. ANC services are also designed to maximise good health outcomes; low maternal and neonatal mortality, low postpartum anaemia, and appropriate birth weight [2,10,11]. Through ANC services a health care provider can inform women about the danger signs and symptoms and can take immediate care to solve the problems[12].

Routine ANC visits, as currently practiced throughout the world, originate from models developed in Europe almost a century ago [13]. According to the European model, women made visits to the clinics or health professionals once a month for the first six months of pregnancy, once every 2–3 weeks for the next two months, and then once a week until delivery. Thus a woman would have about 12 visits during her full term pregnancy. The practice and timing of routine ANC visits were introduced in traditional European model arbitrarily and without evidence of its cost-effectiveness. As the new evidence and technology evolved, many attempts have been made to rationalize the content, frequency and implementation of ANC practices and systematic reviews have examined the effectiveness of individual components of antenatal care programs [14,15,16,17]. Based on a land mark cluster randomized WHO Antenatal Care Trial (WHOACT) in 2001 and careful review of effectiveness of different types of models of maternal health care, the World Health Organization (WHO) recommends an evidence based cost-effective model that pregnant women with uncomplicated pregnancies should receive minimum four ANC visits, with the first visit occurring before 14 weeks of gestation [17,18]. The WHOACT found that a reduction in the number of visits to at least four ANC visits was not inferior to traditional Western ANC packages in the risk of adverse outcomes for mothers and new-borns and could cut cost [17].

In low- and middle-income countries (LMICs), ANC utilization has increased since the introduction in 2002 of the WHO ANC model, known as focused ANC (FANC) or basic ANC, which was a goal orientated approach to delivering evidence-based interventions carried out at four critical times during pregnancy [8]. However, in light of the 2010 Cochrane review [19], which suggested that reduced antenatal visits was in fact detrimental to health, the WHOACT data was reanalyzed by an international group of researchers. It was found that there was an increased risk of fetal death for the women who had reduced numbers of antenatal visits, specifically, for high risk women the risk of fetal death at between 32 and 36 weeks was 80% higher while for low risk women it increased by 50% [20,21]. Based on these findings, the World Health Organization issued a new series of recommendations in 2016 in order to improve quality of antenatal care, reduce the risk of stillbirths and pregnancy complications, and give women a positive pregnancy experience. The recommended interventions span five categories: routine antenatal nutrition, maternal and foetal assessment, preventive measures, interventions for management of common physiologic symptoms in pregnancy, and health system-level interventions to improve the utilization and quality of ANC. The 2016 WHO ANC guidance increases the number of contacts a pregnant woman has with health providers throughout her pregnancy from at least four to at least eight contacts: one contact in the first trimester, two contacts in the second trimester, and five contacts in the third trimester [8].

To make the ANC visits an effective preventive measure, the content and the quality of ANC also need to be monitored. According to WHO, the standard quality of ANC is comprised of three components: (i) assessment, that is history taking, physical examination and laboratory tests, (ii) health promotion, that includes advice on nutrition, planning the birth, information regarding pregnancy, subsequent contraception and breastfeeding, and (iii) care provision that is comprised of tetanus toxoid immunization, psychosocial support and recordkeeping [2]. Although there may be variations in the national strategies about the content of ANC, WHO recommends a core set of services which include blood pressure measurement, tetanus toxoid vaccination, urine testing, iron tablet supplementation, body weight measurement and counseling about danger signs. Yet, in most of the developing countries, a large proportion of women do not receive the minimum four visits [10] and the compliance to minimum level of recommended content for ANC appeared to be unmet due to poor accessibility, inability to afford the costs of seeking care, cultural barriers and lack of knowledge [22–24]. Bangladesh, a South-East Asian developing country, is not an exception. The Ministry of Health and Family Welfare (MH&FW) Bangladesh follow the WHO recommended ANC visits and the core contents of ANC visits.

A good number of studies in Bangladesh have examined the factors associated with the ANC visits and delivery care [25–28]. However, these studies mainly focused on the quantitative coverage of ANC visits, obscuring the content and quality of ANC visits. The content and thus the quality of care may remain poor while the individual coverage of ANC visits could be observed to be high. Previous studies have demonstrated that, in addition to the number of ANC visits, the components covered by ANC visits greatly influence the effectiveness of the ANC services[29,30]. It has also been observed that poor quality ANC influence the utilization of ANC services [31].

Since a woman’s overall reproductive health is determined collectively by the overall coverage and content of ANC visits, it is important to analyze the levels, trends and determinants of ANC coverage and its content with a view to assess the quality of ANC services, so that the government and other stakeholders can take proper action to improve the maternal health and pregnancy outcomes. There are few studies focusing on both the recommended level of ANC visits and its content in developing countries [32–35]. To the best of our knowledge, there is no such study in Bangladesh attempting to identify the factors influencing the coverage and contents of the WHO recommended ANC visits. This study is an attempt to contribute to this end. It aimed to examine the compliance to recommended number of at least eight ANC visits and the extent of adherence to recommended content of ANC visit in Bangladesh. Attempt has also been made to determine the factors influencing the utilization of ANC visits and the content of ANC visits in Bangladesh.

## Methods

### The data and participant

The study used data from the 2014 Bangladesh Demographic and Health Survey (BDHS), which is the seventh in a series of national-level population and health surveys conducted as part of the global Demographic and Health Survey (DHS) program. The detailed of the survey may be seen elsewhere [36]. The 2014 BDHS was a retrospective survey based on two stage stratified-cluster sampling design where each of the seven administrative divisions was treated as strata. The survey covered a nationally representative sample of 17,863 ever-married women aged 15–49 years, who were found in 17,300 randomly selected households. To analyse the coverage and content of ANC visits, we have considered 4,627 women who gave birth in the three years preceding the survey. Among women with two or more live births in the three years preceding the survey, we referred to the last birth only. In the 2014 BDHS, all women who gave birth within three years preceding the survey were asked a number of questions aboutantenatal health care. Information was collected about the person and institution providing ANC (if any), the number of ANC visits, and the items included in the ANC provided.

### Dependent variables

This study considered two outcome variables. These are the frequency of antenatal care (ANC) visits and the content or items of care received during ANC visits. Our second outcome variable relates to receiving the recommended essential components of ANC services during their visits to health personnel. In the 2014 BDHS, data were available for six recommended components or items of ANC services. These included measurement of weight and blood pressure, testing of urine and blood samples, having ultrasound, and counseling about danger sign of pregnancy complications. Information on these six items of ANC content were derived from the response of the question “As part of your antenatal care during this pregnancy, were any of the following done at least once? Was your weight measured? Was your blood pressure measured? …….”. The answers were recorded as Yes or No. It is possible that a single mother may have urine test or blood test or measurement of weight or blood pressure several times during same pregnancy. However, as the mother was asked to report any action at least once, the response for any action was recorded as a single action. On the basis of responses, we have created a composite index of ANC content as our second outcome variable which comprises a simple count of the number of elements of care received. The variable had a minimum value of zero indicating that the women did not receive any ANC services and a maximum value of six indicating that the women received services for all the six elements. Similar type of content index was used by other recent studies [37,38]. The 2014 BDHS did not collect data of timing of first ANC visit.

### Explanatory variables

The study considered mothers’ socio-economic and demographic characteristics as explanatory variables. These included maternal age at birth of the last child (13–19, 20–34, and 35–49), parity (one, two, three and four or more), division (Barisal, Chittagong, Dhaka, Khulna, Rajshahi, Rangpur and Sylhet), place of residence (urban and rural), mother’s educational level (no education, primary, secondary and higher), media exposure (whether women were exposed to television at least once a week or not), wealth quintile of women’s household (the DHS wealth quintile is a composite indicator which divides the households into five categories: poorest, poorer, middle, richer and richest; and were derived using principle component analysis based on information from housing characteristics and ownership of household durable goods)[39], women’s work status (whether in paid employment at the time of the survey or not), decision making power on own health care (self-decision/joint decision with husband, husband alone and other), whether the pregnancy was wanted at thetime (the survey had three categories: ‘pregnancy wanted at that time’, ‘pregnancy wanted later’ and ‘pregnancy not wanted at all’ of which the first two were merged into oneto form the response ‘Yes’ and the last one as ‘No’), number of ANC visits, contraceptive use status (use and not use), ANC provider (the survey had 13 categories of providers which were reduced to no one, skilled and unskilled), place of receiving ANC (home, public, private and NGO).

### Statistical analysis

The data were analysed using univariate (frequency distribution), bivariate and multivariate statistical methods. Frequency distribution was used to describe the characteristics of the overall sample respondents (mothers) across a set of background characteristics. In bivariate analysis simplesummary statistics (either as percentage for the categorical variables or mean for the count variables such as frequency of ANC visits and number of elements received during ANC visits) were obtained for each category of the selected explanatory variables to examine the unadjusted but statistically significant relationship between dependent variables and selected explanatory variables. The statistical significance was tested by a *χ*^2^ (chi-square) test for categorical dependent variable and analysis of variance (ANOVA) for count dependent variable. A *p* value <0·05 was considered statistically significant. Multivariate statistical analyses using generalized linear model (GLM) approach were carried out to ascertain the determinants of the frequency of ANC visits and the number of elements received during ANC visits. Since our response variables are count variables, Poisson regression model i.e. Poisson distribution with a log link is the natural selection [40]. However, the most serious limitation of Poisson regression is that it assumes that the variance of the distribution of the count response variable is equal to its mean which is usually termed as *equidispersion* property. In practical applications, this assumption is often violated as the variance can either be larger (over dispersion) or smaller (under dispersion) than the mean. If the equidispersion assumption is violated, the estimates in Poisson regression model are still consistent but produce invalid inferences about the parameters [41,42]. For over dispesed count variable, Negative Binomial (NB) regression or Generalized Poisson regression are the alternative models for data analysis. Since, in our case, both the response variables viz. frequency of ANC and the number of element of ANC are over dispesed, we employed NB regression model for estimating the regression coefficients. Finally, the risk ratios (RRs) and the corresponding 95% confidence interval were calculated for each category of the predictors. The statistical softwarepackages SPSS 25 is used for all statistical analysis.

## Results

### Characteristics of mothers

Table 1 shows the distribution of mothers according to their background characteristics. At the time of birth of the child, most of the mothers (96%) were under aged 35 years, of which 28% were teenager of age 13–19, and the average age of the mothers were 23.6 years. Most of the mothers had parity 1 or 2 (70%), had primary or above level education (86%), living in rural area (73.9%), currently using family planning method (66.5%), having no employment (76.3%), and could take decision on health care by themselves or jointly with their husbands (62%). More than half (57%) of mothers were from two large administrative divisions, namely Dhaka (35%) and Chittagong (22%), and the proportion of mothers varied from 5.8% to 10% across the other five divisions. About 49% of the mothers were reported to have had mass media (i.e. TV) exposure for at least once in a week. About 11% of the mothers reported that their pregnancy was unwanted. More than half (53%) of the mothers received ANC services from private health facilities, followed by the public health facilities (28%), and the nongovernmental organizations (NGO) health facilities (9%). About 10% of mothers received ANC services at home (Table 1).

**Table 1:** Percentage distribution of women, percentage of women who received four or more ANC visits, and un-adjusted and adjusted odds ratio with 95% confidence interval (95% CI) of 4 or more ANC visits according to background characteristics, Bangladesh 2014.

Characteristics	Number of women (%)	% of women who had ≥8 ANC visits	p-value^a^	Mean of frequency of ANC visits	p-value^b^	Mean of number of ANC items	p-value^b^
**Total**	4627 (100)	6.1		2.70		3.29	
**Women age at birth of child**			0.043		0.001		0.010
<20 years	1293 (27.9)	6.0		2.68		3.30	
20–34 years	3132 (67.7)	6.4		2.75		3.34	
35–49 years	202 (4.4)	2.5		2.07		2.83	
**Parity**			<0.001		<0.001		<0.001
1	1847 (39.9)	7.8		3.09		3.69	
2	1392 (30.1)	7.2		2.86		3.38	
3	748 (16.2)	3.2		2.35		3.01	
4+	639 (13.8)	2.5		1.62		2.25	
**Administrative division**			0.006		<0.001		<0.001
Barisal	268 (5.8)	4.9		2.38		3.10	
Chittagong	1010 (21.8)	5.6		2.41		3.26	
Dhaka	1635 (35.3)	7.4		2.95		3.58	
Khulna	371 (8.0)	8.1		3.33		3.70	
Rajshahi	464 (10.0)	5.4		2.48		2.93	
Rangpur	450 (9.7)	6.0		3.07		3.09	
Sylhet	429 (9.4)	2.6		1.89		2.60	
**Place of residence**			<0.001		<0.001		<0.001
Urban	1209 (26.1)	11.2		3.67		4.14	
Rural	3418 (73.9)	4.3		2.36		2.99	
**Mother’s education**			<0.001		<0.001		<0.001
No education	655 (14.2)	1.7		1.50		1.98	
Primary	1293 (27.9)	3.7		2.02		2.63	
Secondary	2208 (47.7)	7.1		3.08		3.73	
Higher	471 (10.2)	14.4		4.45		4.85	
**Mass media exposure (TV)**			<0.001		<0.001		<0.001
Not at all	1928(41.7)	3.2		1.87		2.40	
Less than once a week	421 (9.1)	5.0		2.44		2.97	
At least once a week	2278 (49.2)	8.8		3.45		4.10	
**Wealth index**			<0.001		<0.001		<0.001
Poorest	1003 (21.7)	1.6		1.55		1.81	
Poorer	876 (18.7)	5.0		2.06		2.65	
Middle	881 (19.0)	5.6		2.60		3.28	
Richer	955 (20.6)	6.3		3.17		3.96	
Richest	912 (19.7)	12.6		4.18		4.82	
**Contraceptive use**			0.432		0.056		0.005
Not use	1549 (33.5)	6.0		2.55		3.16	
Use	3078 (66.5)	6.2		2.77		3.35	
**ANC Provider**			<0.001		<0.001		<0.001
Skilled	2902 (62.7)	7.5		3.34		4.34	
Unskilled	1725 (37.3)	3.9		1.62		1.52	
**Place of receiving ANC**			<0.001		<0.001		<0.001
Home	454 (9.8)	8.4		3.80		3.14	
Public	1291 (27.9)	6.0		3.31		4.07	
Private	2470 (53.4)	4.9		2.00		2.71	
NGO	413 (8.9)	11.7		3.76		4.46	
**Employment status**			0.645		0.054		<0.001
Not employed/housewife	3532 (76.3)	5.8		2.76		3.04	
Employed	1095 (23.7)	6.2		2.50		3.36	
**Decision on health care**			0.048		0.031		<0.001
Woman alone	513 (11.1)	8.2		3.03		3.64	
Woman and husband	2336 (50.5)	5.8		2.77		3.39	
Husband alone	1375 (29.7)	5.3		2.42		2.93	
Other	403 (8.7)	7.4		2.80		3.45	
**Husband’s education**			<0.001		<0.001		<0.001
No education	1105 (23.9)	2.2		1.73		2.25	
Primary	1387 (30)	5.6		2.38		2.88	
Secondary	1469 (31.7)	6.7		3.01		3.79	
Higher	666 (14.4)	12.7		4.27		4.76	
**Pregnancy was wanted at time**			<0.001		0.001		<0.001
Yes	4121 (89.1)	6.6		2.79		3.36	
No	506 (10.9)	2.0		1.94		2.67	
**Frequency of ANC visits**							<0.001
0	991(21.4)					0.00	
1	833 (18.0)					3.30	
2	748 (16.2)					4.02	
3	614 (13.3)					4.26	
4	474 (10.2)					4.59	
5+	967 (20.9)					4.93	

^**a**^ p-values are based on Chi-square test for testing variation among proportions

^**b**^ P-values are based on Analysis of Variance (ANOVA) and F-test for testing the variation among means.

### Level of utilization of ANC services

**Fig 1:**
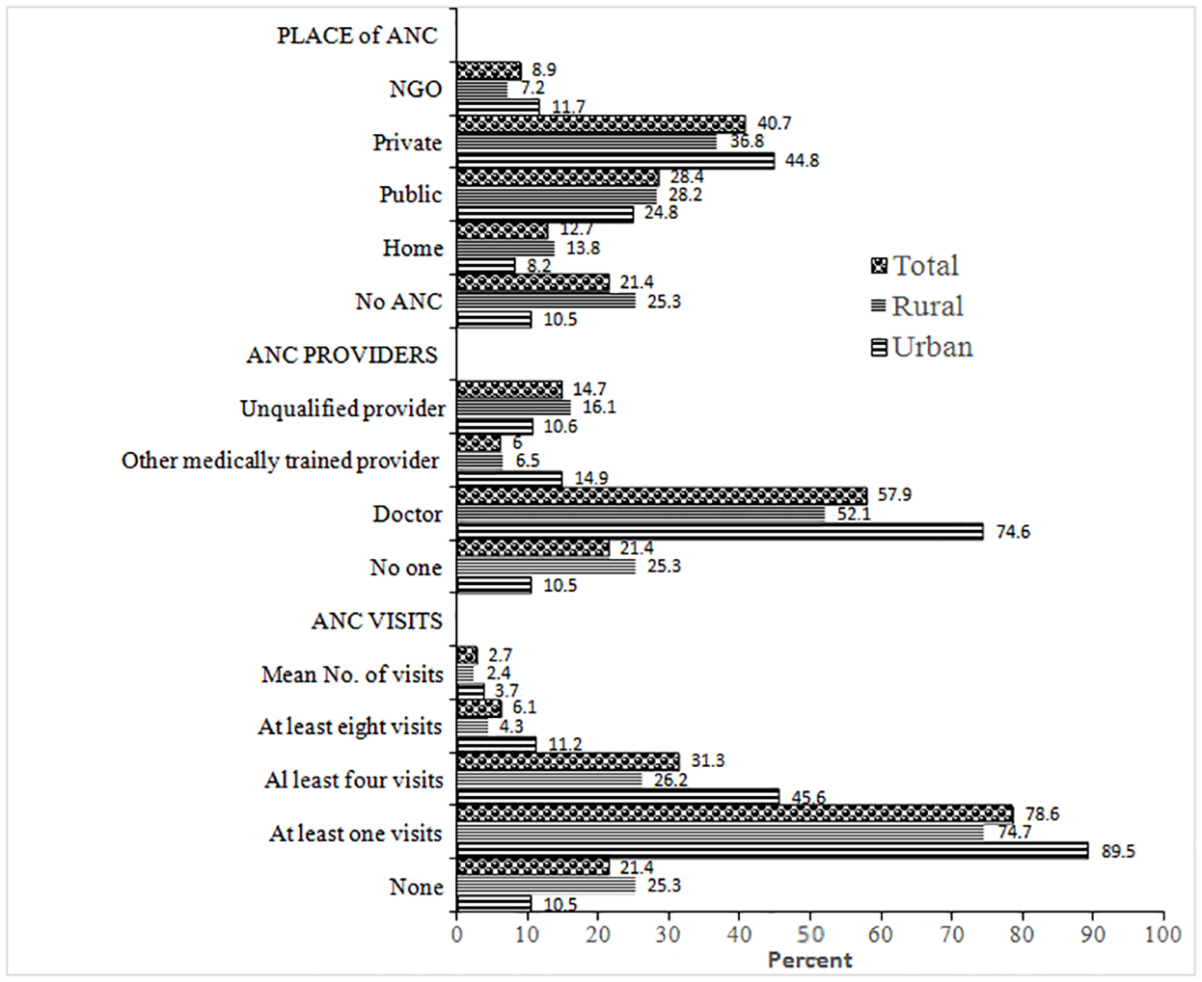
Percentage distribution of women by antenatal care (ANC) visits, ANC provider and place of ANC services received according to urban/rural place of residence, Bangladesh 2014.

Fig 1 shows the distribution of mothers according to the pattern of utilization of ANC services with urban-rural differentials. The analysis shows that about 79% of the mothers with a birth in the three years preceding the survey had at least one ANC visits;31.3% had at least four ANC visits, while only 6% had updated WHO recommended at least eight ANC visits. About one-fifth (21%) of the mothers did not receive any ANC visits. As expected, mothers from urban area were 2.6 times more likely to use eight or more ANC visits than the mothers from rural area (11.2% vs 4.3%). However, one in every five (21.4%) mothers never received any ANC visits. Mothers from rural area were 2.4 times more like to have no ANC visits than the mothers from urban area (25.3% vs. 10.5%). On average mothers received less than 3 visits. The average number of visits among urban mothers was 3.7, while for rural mothers it was 2.4. Those who received ANC visits, most of them received ANC from medically trained providers (Fig 1). Overall about two-thirds of mothers (64%) received ANC from medically trained provider– 58% from qualified doctor and 6% from other medically trained providers including nurse, midwife, paramedic, family welfare visitor (FWV), community skilled birth attendant (CSBA), or sub-assistant community medical officer (SACMO). Urban mothers were more likely to receive ANC from medically trained providers than the mothers from rural area (89.5% vs. 58.6%). Overall, two-third mothers received ANC services from health facilities.

The analysis shows that the number ofantenatal visits during a pregnancy ranged from 1 to20, with a mean of less than 3 visits (2.7 visits), a median of 2.0 visits, and a variance of 6.2 visits, indicating that the distribution has over dispersion. There were a few cases with high frequencies, but majority (69%) had less than four visits, makingA J-shaped distribution of the frequency of ANC visits.

### Differentials of frequency of ANC visits: Bivariate analysis

Table 1 presents the results of the bivariate analysis of the summary statistics of the frequency of ANC visits, such as the mean of the frequency of ANC visits and the proportion of at least eight ANC visits as recommended by WHO, across a set of selected socio-economic and demographic characteristics, ANC provider and place of ANC visits. The analysis indicate that maternal age at birth of child, parity of mothers, administrative region, place of residence, education of mothers and their husbands, mass media exposure, wealth status, decision on healthcare, pregnancy wanted, ANC provider and the place of ANC visits have significant association with the frequency of ANC visits. Mothers aged20-34 years old at the time of birth of the child were more likely to have eightor more ANC visits and higher mean of the frequency of ANC visits than their youngers (aged <20 years) and older (aged 35 and above) counterparts. Mother’s parity showed significant negative association with both the mean of ANC visits and the proportion of at least eight ANC visit. The coverage of eight or more ANC visits was found to be 7.8% among the mothers with parity one, compared to 2.5% among the mothers with parity four or more. Among the six administrative divisions, Khulna showed the higher proportion of at least eightANC visits as well as higher mean of ANC visits and Sylhet the lowest. Urban mothers were more likely to have eightor more ANC visits and higher mean number of ANC visits than the rural mothers. Mothers’ education and wealth status as well as husbands’ education showed significant positive association with the mean of ANC visits and the proportion of mothers who had eight or more ANC visits. For example, the mean of ANC visits was found to be 1.5 visits among the mothers with no education compared to 4.5 ANC visits among mothers with higher level of education. Media (TV) exposure showed significant positive association with mean of ANC visits and the proportion of having eight or more ANC visits. Mothers with wanted pregnancy were more likely to have eight or more ANC visitsand higher mean of ANC visits thanthe mothers with unwanted pregnancy (6.6% vs. 2.0%). Mothers’ decision making power on their own healthcare, ANC service provider and the place of receiving ANC services showed significant association with mean of ANC visits and the proportion of mother who had eight or more ANC visits.

### Determinants of frequency of ANC visits: Multivariate analysis

The foregoing bivariate analysis presents unadjusted effects of the explanatory variables on frequency of ANC visits. To identify the significant adjusted or net effect of an explanatory variable on frequency of ANC visits, we employed multiple Negative Binomial regression model. The net effect of an explanatory variable was measured by the odds ratio after controlling the effects of all other explanatory variables. The results for only significant predictors are presented in the Table 2.

**Table 2:** Results of the multivariable generalized linear regression analysis with Negative Binomial log link to identify the determinants of frequency of ANC visits, Bangladesh 2014.

Factors	Estimated regression coefficient (β)	p-value	Odds ratio (OR)	95% CI of OR	
				Lower	Upper
**Parity**					
1	0.175	0.030	1.191	1.018	1.393
2	0.139	0.058	1.149	0.995	1.327
3	0.122	0.100	1.129	0.977	1.306
4+ (ref)	0^a^		1		
**Region**					
Barisal	0.176	0.118	1.193	0.956	1.488
Chittagong	0.060	0.444	1.062	0.910	1.240
Dhaka	0.261	0.001	1.298	1.119	1.506
Khulna	0.323	<0.001	1.381	1.155	1.652
Rajshahi	0.139	0.130	1.149	0.960	1.374
Rangpur	0.437	<0.001	1.548	1.296	1.848
Sylhet (ref)	0^a^		1		
**Place of residence**					
Urban	0.149	0.002	1.161	1.056	1.276
Rural (ref)	0^a^		1		
**Woman education**					
No education (ref)	0		1		
Primary	0.197	0.004	1.218	1.064	1.394
Secondary	0.397	<0.001	1.488	1.293	1.711
Higher	0.462	<0.001	1.588	1.313	1.920
**Media exposure (TV)**					
Not at all (ref)	0^a^		1		
Less than once a week	0.122	0.076	1.129	0.988	1.291
At least once a week	0.211	<0.001	1.235	1.121	1.360
**Wealth index**					
Poorest (ref)	0^a^		1		
Poorer	0.145	0.019	1.156	1.025	1.305
Middle	0.154	0.021	1.167	1.023	1.330
Richer	0.286	<0.001	1.331	1.159	1.529
Richest	0.393	<0.001	1.481	1.261	1.740
**ANC Provider**					
Skilled	0.877	<0.001	2.403	2.200	2.625
Unskilled (ref)	0^a^		1		
**Place of receiving ANC**					
Home	0.383	<0.001	1.467	1.250	1.721
Public	-0.361	<0.001	0.697	0.608	.800
Private	-0.974	<0.001	0.378	0.331	.431
NGO (ref)	0^a^		1		
**Husband’s education**					
No education (ref)	0^a^		1		
Primary	0.149	0.001	1.161	1.041	1.231
Secondary	0.178	0.015	1.194	1.080	1.346
Higher	0.386	<0.001	1.471	1.259	1.718

^a^ Set to zero because it is reference category (ref).

The regression analysis identified parity, administrative division, place of residence, mothers’ and fathers’ educational level, media exposure, wealth quintile, ANC provider and place of receiving ANC as significant predictors of frequency of ANC visits in Bangladesh (Table 2). Mothers with parity 1 were found to be 1.19 times more likely to have at least one ANC visits than the mothers with 4 or more parity (OR = 1.19, (5% CI: 1.018–1.28). Among the six administrative divisions, mothers who were living in Rangpur division had 1.5 times higher odds of receiving ANC visits compared to mothers who were living in Sylhet division (OR = 1.54, 95% CI:1.29–1.84). Mothers from Khulna and Dhaka divisions also had 1.4 and 1.3 times higher odds of receiving ANC visits, respectively, than the mothers from Sylhet division. Mothers from urban area were 1.16 times more likely to receive ANC visits than their counterparts in rural area (OR = 1.16; 95% CI: 1.06–1.28). Education levels of mother and their husbands showed significant positive association with frequency of ANC visits. Mother with higher level of education were 1.59 times more likely to have ANC visits than the women with no education (OR = 1.59, 95% CI:1.31–1.92). The odds of receiving any ANC visits among the richest mothers were 1.48 times higher than the mothers who were poorest(OR = 1.48, 95% CI: 1.26–1.74). Mothers having media (TV) exposure for at least once a week were found to be 1.24 times more likely to have ANC visits than the women with no media exposure (OR = 1.24; 95% CI: 1.21–1.36). Mothers who obtained ANC services from medically trained health personnel had2.4 times higher odds of receiving ANC visits than the mothers who obtained ANC services from unskilled health personnel (OR = 2.40, 95%CI:2.20–2.63). Mothers who received ANC services from public or private health institutions were 30% and 62%, respectively, less likely to have ANC visits than the mothers who received ANC services from non-government organizations (NGO).

### Content of ANC services

**Fig 2:**
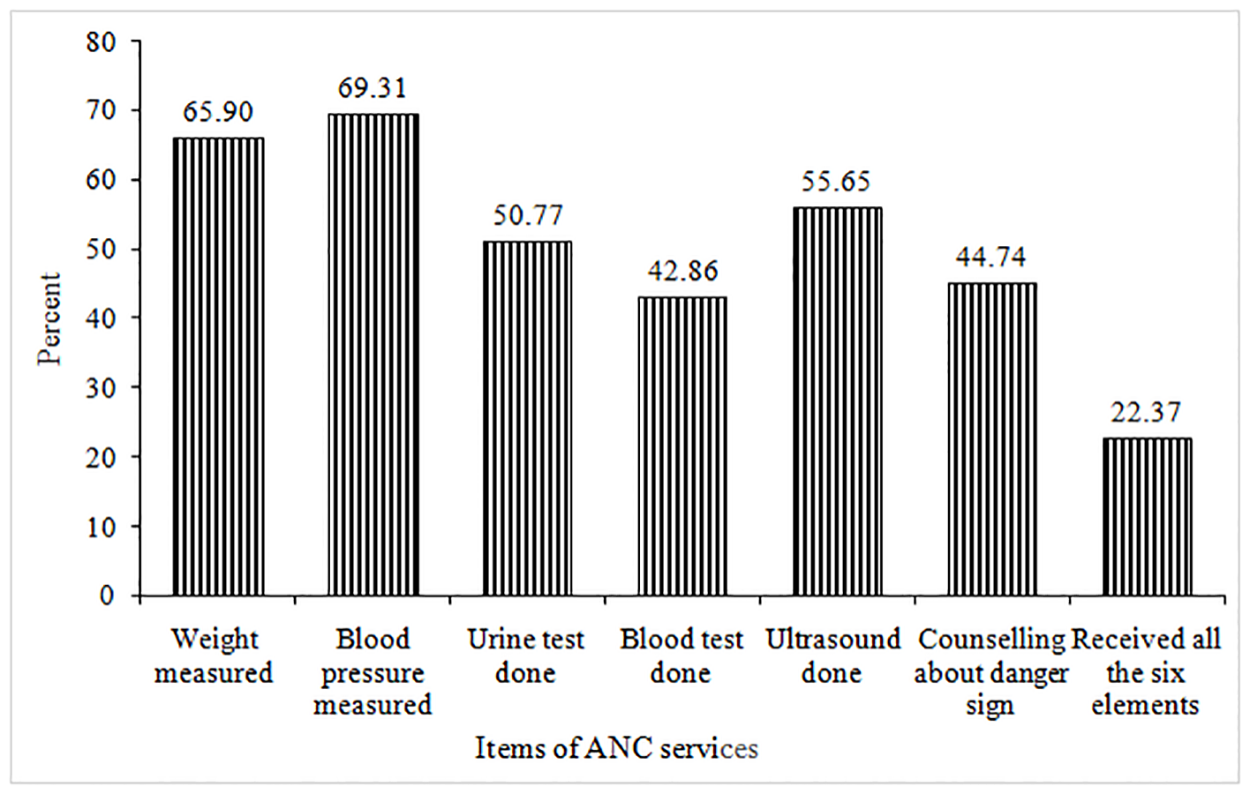
Percentage of women who received recommended elements or items of ANC services received during last pregnancy, Bangladesh 2014.

Fig 2 presents the percentage of mothers receiving recommended components or items of ANC services in Bangladesh. As mentioned earlier, the 2014 BDHS collected data on the following six essential components of ANC services: measurement of weight and blood pressure, testing of urine and blood samples, performing ultrasound and informing and counseling about danger sign of pregnancy complications. Our analysis shows that, on average, only 21% of mothers received all the six selected elements of ANC services. Considering the individual components, blood pressure measurement was the most common item received by 69% mothers, closely followed by weight measurement by 66% mothers. More than half (56%) of mothers were reported to have ultrasonic scan, 51% had urine testing, and 45% mothers reported that they were informed and advised about pregnancy complications during the ANC visit. The least received component was the blood testing done by 43% of mothers (Fig 2).

**Fig 3:**
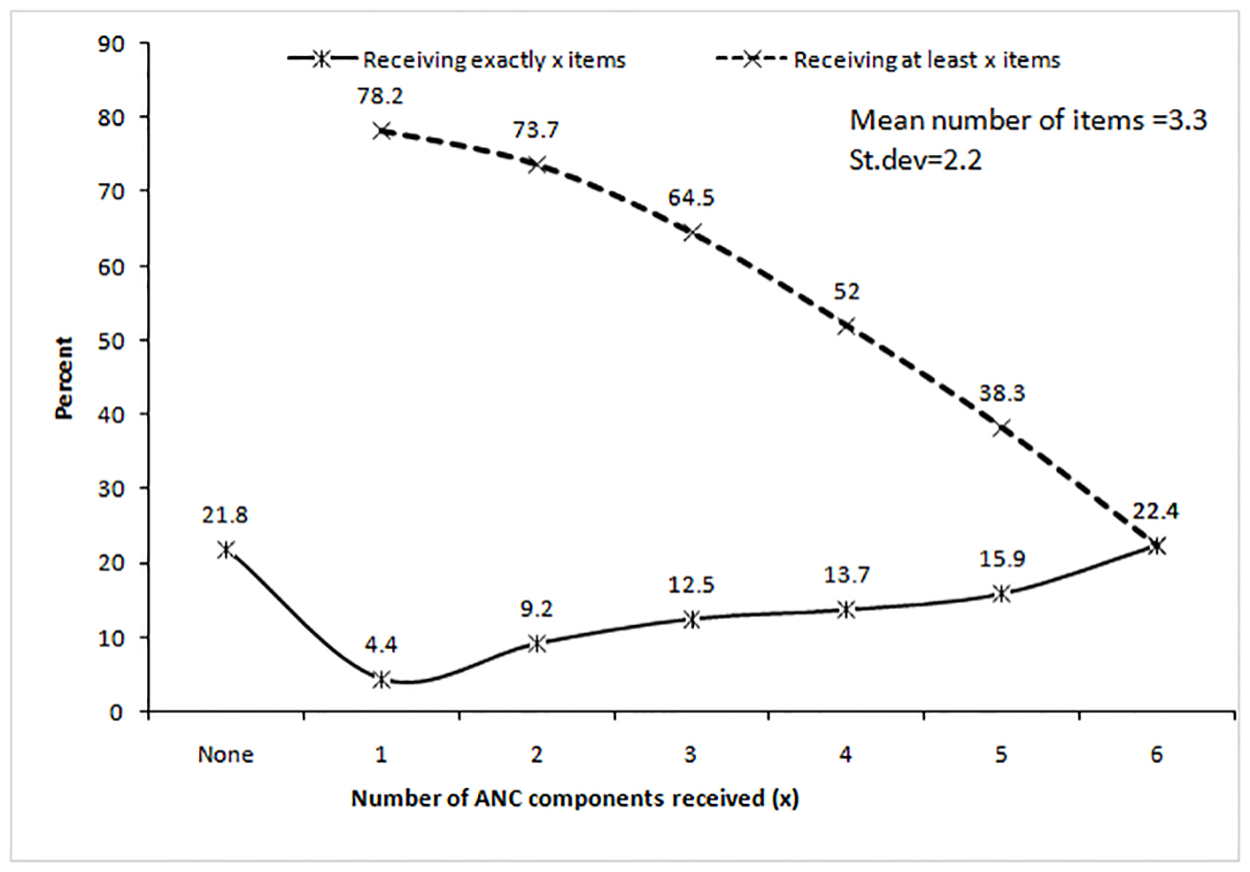
Distribution of the number of ANC components or items received by the mothers.

Fig 3 presents the distribution of the number of items of the ANC services received by mothers during ANC visits. The number of items received by the mothers varies from 0 to 6 items, with a mean of 3.29 items, and a variance of 4.9 items, indicating that the distribution is over dispersed. About 79% mothers received at least one of the six selected items of the ANC services and the rest 21% received no ANC services during their last pregnancy. About 5% mothers received only one of the six items. The percentage of mothers hold on increasing with the increase of the number of the items. About 14% mothers received four out of six items, while 16% received five out of six items and 22% received six out of six items.

### Differentials of the receipt of ANC items: Bivariate analysis

**Fig 4:**
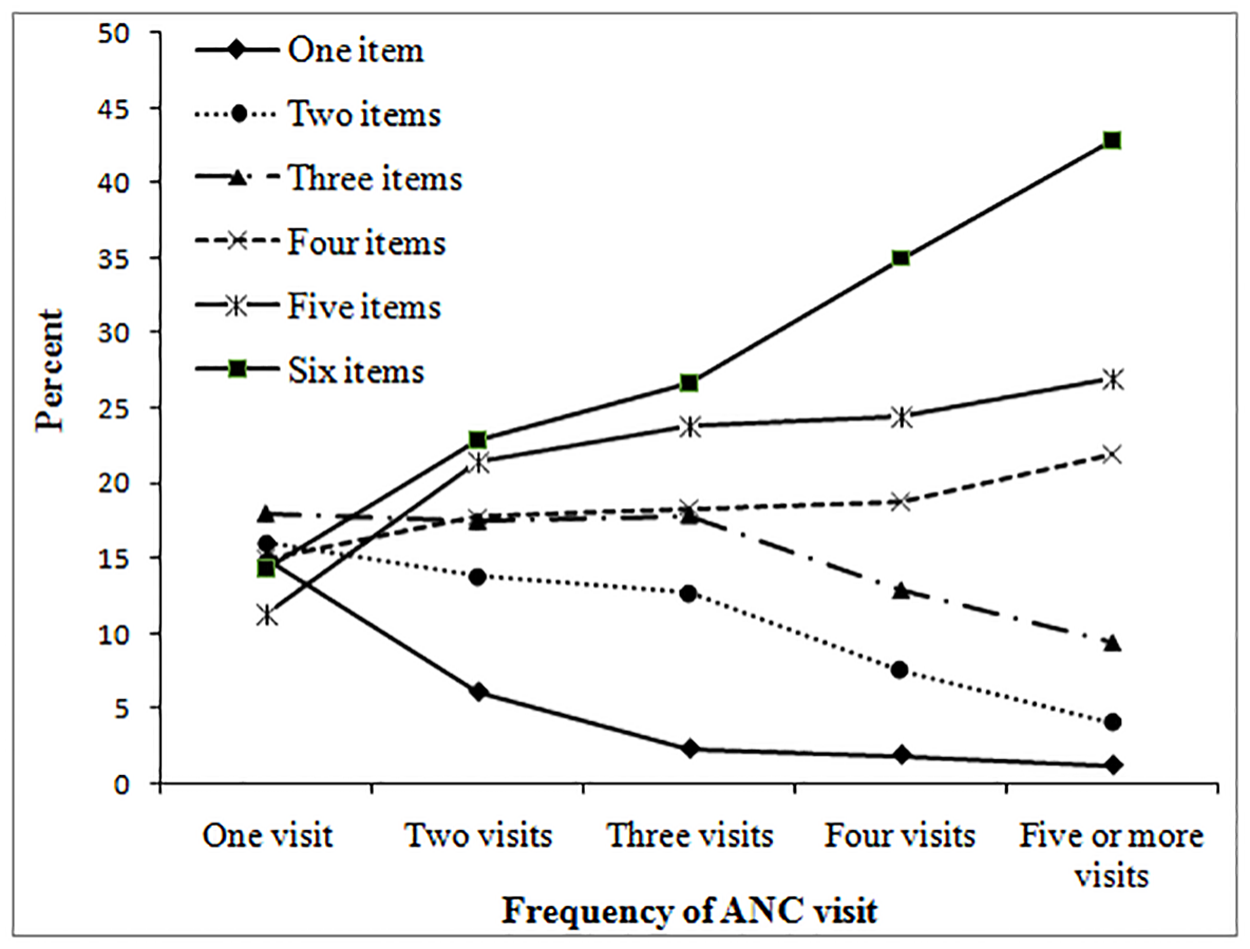
Percentage of number of items of ANC services by frequency of ANC visits.

The results of the bivariate analysis using mean of the number of items of ANC services across a set of explanatory variables indicate that all the selected socio-economic and demographic characteristics of mothers, ANC provider, place of ANC visits and the number of ANC visits have significant association with the number of items of ANC services. The mean of items of ANC services received by the mothers during their ANC visits vary significantly across mothers age, parity, education, husband’s education, wealth status, administrative region, place of residence, mass media exposure, decision on health care, wanted pregnancy, ANC providers, place of ANC visits and the number of ANC visits. The number of items of ANC services shows significant positive association with the frequency of ANC visits, as the number of ANC items received increased with the increase of frequency of ANC visits. For example, mothers with only one ANC visits had received on an average 3.3 items of ANC services, compared to almost 5 items on average among the mothers with five or more ANC visits (Table 1). Fig 4 presents more detailed analysis of the relationship between each number of items of ANC contents and the frequency of ANC visits. It is evident that the likelihood of receiving higher number of items of ANC content increase monotonically with the increase of frequency of ANC visits. The proportion of mothers having six items of ANC content has increased from 14%, to 43% with the increase of ANC visits from only one visits to five or more ANC visits. Similar patterns have been observed for having five and four items of ANC content. However, the likelihood of receiving of only one item or two items or three items showed declining trends with the increase of number of ANC visits.

### Determinants of the contents of ANC visits: Multivariate analysis

Table 3 presents the results of multiple regression analysis predicting the utilization of the items of the content of ANC visits by a mother during her pregnancy. The results indicate that mother’s parity, education, wealth status, mass media (TV) exposure, place of residence, region of residence, wanted pregnancy, number of ANC visit, ANC provider and place of receiving ANC services as significant predictors of receiving the items of ANC services. Mother’s education showed strong positive association with the number of elements received during ANC visits. Mothers who had higher level of education were 1.4times more likely to receive the items of ANC content than the mothers who had no education (OR = 1.389; 95% CI: 1.155–1.666). Similar results were found for father’s education. Wealth status of mothers also showed significant positive association with the receipt of ANC services. Mothers in the richest group compared to those in the poorest group were 1.5 times (OR = 1.513; 95% CI: 1.299–1.763) more likely to receive items of ANC content. Similarly, mothers in middle and richer group were, respectively, 1.3 and 1.5 times more likely to receive the items ofANC contents relative to women in poorest group. Mothers with low parity, particularly with parity 1 had higher likelihood of using the elements of ANC services than the mothers with parity four or more (OR = 1.078, 95% CI:1.014–1.158). Mothers from urban areas were 1.4 times more likely to receive the items of ANC services than their rural counterparts (OR = 1.351; 95% CI: 1.104–1.496). Mothers from Dhaka and Khulna divisions, respectively, had20% (OR = 1.203; 95% CI:1.108–1.460) and 14% (OR = 1.144; 95% CI:1.059–1.356) higher odds of receiving items of ANC content than the mothers from Sylhet division. Mothers who had TV exposure for at least once a week had 10% (OR = 1.108; 95% CI:1.048–1.165) higher odds of receiving items of ANC services compared to mothers who had no TV exposure. Mothers were found to have at least 10% higher odds of receiving items of ANC services if the pregnancy was intended at the time (OR = 1.102; 95% CI: 1.012–1.142).

**Table 3:** Results of the multivariable generalized linear regression analysis with Negative Binomial log link to identify the determinants of utilization of contents of ANC visits, Bangladesh 2014.

Factors	Estimated regression coefficient (β)	P-value	Odds Ratio	95% CI of OR	
				Lower limit	Upper limit
**Parity**					
1	0.085	0.012	1.089	1.014	1.153
2	0.045	0.062	1.046	0.983	1.115
3	0.053	0.124	1.054	0.986	1.127
4+ (ref)	0^a^		1.000		
**Administrative division**					
Barisal	0.035	0.546	1.035	0.839	1.278
Chittagong	0.055	0.457	1.056	0.914	1.221
Dhaka	0.185	0.001	1.203	1.108	1.460
Khulna	0.134	0.037	1.144	1.059	1.356
Rajshahi	0.004	0.074	1.004	0.848	1.188
Rangpur	0.139	0.106	1.149	0.971	1.360
Sylhet (ref)	0^a^		1.000		
**Place of residence**					
Urban	0.301	0.034	1.351	1.104	1.496
Rural (ref)	0^a^		1.000		
**Mother’s education**					
No education (ref)	0^a^		1.000		
Primary	0.109	0.016	1.115	1.021	1.219
Secondary	0.231	<0.001	1.260	1.120	1.573
Higher	0.327	<0.001	1.387	1.155	1.666
**Media (TV) exposure**					
Not at all (ref)	0^a^				
Less than once a week	0.038	0.240	1.039	0.975	1.107
At least once a week	0.103	<0.001	1.108	1.048	1.165
**Wealth index**					
Poorest (ref)	0^a^		1.000		
Poorer	0.188	0.003	1.207	1.074	1.356
Middle	0.240	<0.001	1.271	1.120	1.441
Richer	0.374	<0.001	1.453	1.274	1.659
Richest	0.414	<0.001	1.513	1.299	1.763
**Husband’s education**					
No education (ref)	0^a^		1.000		
Primary	0.056	0.152	1.057	0.886	1.051
Secondary	0.089	0.042	1.093	1.065	1.211
Higher	0.220	0.004	1.246	1.073	1.448
**Pregnancy wanted**					
Yes	0.097	0.039	1.102	1.010	1.142
No (ref)	0^a^		1.000		
**ANC Visits**					
Less than 8 visits (ref)	0^a^		1.000		
8 or more visits	0.234	0.001	1.264	1.102	1.450
**ANC Provider**					
Skilled	1.103	<0.001	3.013	3.050	3.624
Unskilled (ref)	0^a^		1.000		
**Place of receiving ANC**					
Home (ref)	0^a^		1.000		
Public	0.064	0.038	1.066	1.012	1.201
Private	-0.269	<0.001	0.764	0.482	0.856
NGO	0.035	0.665	1.035	0.844	1.213

Note:

^a^ Set to zero because it is reference category (ref).

ANC related factors such as number of ANC visits, ANC provider and place of receiving ANC services appeared as the significant predictors for receiving ANC services. Mothers seen by skilled health workers had 3.0 times higher odds of receiving items of ANC services than the mothers seen by unskilled providers (OR = 3.013; 95% CI:3.050–3.624). Mothers who had eight or more ANC visits were 1.3 times (OR = 1.264; 95% CI: 1.102–1.450) more likely to receive items of ANC services than the mothers who had less than eight ANC visits. Mothers who visited private sector health facilities for ANC services were found to have 24%(OR = 0.764; 95% CI: 0.482–0.856) less odds of receiving items of ANC services than the mothers who received ANC at home. On the other hands, mothers who received ANC services from public sector health facilities had 7% higher odds of receiving items of ANC services relative to mothers who received ANC at home (OR = 1.066, 95% CI:1.012–1.201).

## Discussion

The findings of the study revealed that nearly eighty percent (78.6%) of the mothers with a birth in three years preceding the survey (2012 to 2014) received at least one ANC visits during pregnancy. However, only 6% of the mothers received the WHO recommended eight or more ANC visits and 31% received at least four ANC visits. Thus, according to the updated guidelines of WHO, a vast majority (94%) of the mothers did not receive at least eight ANC visits, while 69% did not receive the minimum required four ANC visits as recommended previously. This indicates a very unsatisfactory compliance with the WHO recommended level of ANC visits in Bangladesh. It is worth mentioning here that the assessment conducted by this study on the extent of adherence to recommended practices of ANC is based on data collected in 2014 when the national practice of ANC in Bangladesh was based on the WHO previous guidelines of at least four ANC visits. In 2010, the Health, Population, and Nutrition Sector Development Program (HPNSDP) of the Ministry of Health (MoH) of Bangladesh set a target to achieve that at least 50% of pregnant women receive at least four ANC visits by 2016[43]. Our analysis revealed that the target is still far reaching. It is, however, encouraging to note that the utilization of four or more ANC visits shows an increasing trend in Bangladesh over the last one decade. A comparison of the 2014 BDHS results with the 2004 BDHS results indicate that the percentage of women who had no ANC visit has declined from 42% in 2004 to 21% in 2014. At the same time, the percentage of pregnant women who made four or more antenatal visits has increased, from 17% in 2004 to 31% in 2014[36].

Based on available information, our analysis revealed that nearly one-fifth (22%) of the mothers with a birth in three years preceding the survey date received all the six selected ANC services. On average mothers received ANC services on 3.6 items out of six items. The analysis also shows that the commonest component of ANC offered inBangladesh are measurement of blood pressure reported by 69% mothers and the body weight reported by 66% mothers. Blood test was the least received item (43%).

This study analyzed the factors affecting the frequency of ANC visits and the factors affecting the receipt of the number of elements of ANC services. The results indicate that mother’s education, wealth status, parity, media exposure, place of residence, region of residence, husband’s education, ANC provider and place of receiving ANC as significant predictors of both frequency of ANC visits and the receipt of the items or element of ANC services in Bangladesh. In addition, planned pregnancy and the frequency of ANC visits were found to be significant predictors of the receipt of items of ANC services.

Our analysis indicates significant positive association between frequency of ANC visits received and the utilization of higher number of items of ANC services. Among the mothers who had only one ANC visits, 14% of them were found to have all the six selected items of ANC services. However, the proportion of receiving all the six selected items of ANC contents increased to 43% for the mothers who had five or more ANC visits (Fig 4). After controlling the other potential confounders of receipt of the items of ANC services, the frequency of ANC visits remain significant predictor of the receipt of the items of ANC visits. Mothers who had eight or more ANC visits were found to have26% higher odds of receiving items of ANC services (Table 3). Similar findings were reported by many recent studies in different settings [34,38,44,45]. This study also documents ANC service providers and the place of ANC visits as strong significant predictors of both receipt of elements of ANC services and the frequency of ANC visits. It was observed that the odds of receiving the items of ANC contents was three times higher for mothers who attended skilled providers of ANC services (i.e. qualified doctor, nurse/midwife/paramedic, family welfare visitor, community skilled birth assistant and sub-assistant community medical officer), compared with the unskilled (health assistant, family welfare assistant and community health care provider) providers. Similarly, the odds of frequency of ANC visits were found to be two times higher for skill provider compared to unskilled providers. The odds of receiving the items of ANC services were found to be higher among the mothers who received ANC services from public sector health facilities compared with the mothers who received ANC services from home. The odds of frequency of ANC visits and receiving items of ANC services also found higher among the mothers who visited NGO (non-government organization) health facilities. This may be due to the fact that both public and NGO health facilities are more accessible and less costly. Our findings of the positive impact of the program factors (skill provider and type of provider) on content or frequency of ANC visits are consistent with the finding of many recent studies[30,31,34,35].

Mother’s levels of educational attainment were found to have significant positive association with the frequency of ANC visits and the receipt of the items of ANC services in Bangladesh. This finding of a strong effect of maternal education on optimal use of ANC services is consistent with findings from other parts of the world [32,34,35,38,46]. There are several pathways through which maternal education might affect their health seeking behavior, leading to greater utilization of optimum level health care. Educated mothers are more likely to have knowledge about maternal health care and the most appropriate services for their needs[28,29,47]. Educations also increase mother’s decision-making power within the household [47] and builds greater confidence and capability to make decisions regarding their own health[12]. Mothers’ education also foster new values and attitudes that is favorable to greater use of modern health care[48,49]. Father’s education also showed similar positive association with the greater use of four or more ANC visits and the contents of ANC visits. Therefore, policies aimed at improving level of education to at least secondary level, particularly among female, are likely to improve both quantity and quality of the ANC use. For Bangladesh this is particularly very important, where 27% women never attended school, only 12% women completed secondary and above level of education [36].

The present study findings revealed that the household wealth status is a significant predictor of both the frequency and the items of ANC services, which is in agreement with other studies findings conducted in Bangladesh and elsewhere [34,35,38,50,51]. In a country like Bangladesh, where over one third of the population living in poverty and another one third living just above the poverty level [52], economic factors could impact the health seeking behaviour of women in many ways. Without an overall improvement in the standard of living, efforts for further improvement of effective utilization of maternal health care would not bring any fruitful results.

The mass-media exposure of mothers appeared as significant determinant maternal health seeking behaviour. Mothershaving media exposure (i.e. television) were more likely to know theproblems associated with pregnancy and importance of optimal use of ANC services. As the government of Bangladesh is disseminating important health messages through, television, it is possible that such mass-media campaigns may have had a positive impact on greater utilization of both frequency and the content of ANC services.

The current study found significant variations in frequency of ANC visits received and receiving items of ANC services across the seven administrative divisions. Sylhet divisions showed lowest performance in both frequency of ANC received and the items of ANC services received, while Khulna division showed the highest performance of both the ANC indicators, closely followed by Rangpur and Dhaka division. Dhaka division includes the megacity Dhaka which is the capital city of Bangladesh, and might be associated with higher availability of good quality ANC services. The observed regional variations in utilization of ANC services may be due to variation in health seeking behaviour, availability, accessibility and quality of services. Further study is needed to identify the factors responsible for belter performance in Khulna and Rangpur division and lowest performance in Sylhet division.

Women living in rural areas were found to be less likely to receive less number of ANC visits and items of ANC services compared to their urban counterparts. Many previous studies also reported lower utilization of ANC services among rural women[34,35,38,53,54]. Lower rate of use of ANC services among rural women might be linked to the lower socio-economic conditions of rural women, limited health care services and less accessibility to health care facilities due to poor or even no transportation facility in Bangladesh.

Mothers’ parity showed a negative association with utilization of ANC services. As the number of children a woman have increased, utilization of optimal level ANC services becomes less likely. The result is in agreement with the results from other studies [34,38,52]. Because of different complications for the first pregnancy, greater use of ANC services for the first one or two pregnancy might be related to factors such as fear of unknown complications and excitement over a first baby [54].

Unwanted pregnancy was found to be associated with the use of less items of ANC contents in Bangladesh compared to wanted pregnancy. Similar finding were reported by studies elsewhere [45,55]. The finding indicates that couples are usually less careful about receiving ANC services in case of unwanted pregnancy. Health providers should be trained how to identify the unwanted pregnancy and provide culturally-appropriate support and care to women with unwanted pregnancy [12].

This study has both strength as well as some limitations. The strength lies in the fact that it is based on a large data set of the nationally representative ‘Demographic and Health Survey’ (DHS) that used “validated questionnaires and methodology” [56]. As a result, the the findings are generalizable to the national as well as subnational levels. The study is the first of its kind in Bangladesh to evaluate the level of compliance with the WHO recommended number of ANC visits and the content of ANC visit, and thus assessing the quality of ANC visits. The study findings may have important policy implication for further improvement of ANC services. It may also have potential to contribute in the literature. Nevertheless, the study is not free from limitations. The findings are likely to suffer from selection biases, because the data used in the study is cross sectional in nature which was obtained through retrospective interview of a selected group of women who had a live birth within three years of the survey. While collecting ANC related information, the survey excluded other types of pregnancy outcomes as well as the mothers who had died during pregnancy or delivery. As a result the health seeking behaviours of those excluded mothers remained unknown, although the health seeking behaviour of those mothers may have important policy implications for further improvement of the maternal health outcomes. Moreover, the retrospective nature of the data may have introduced recall biases that may not correctly presents the health seeking behaviours of mothers. Due to data limitation, this study mainly focused on the socio-economic and demographic determinants of ANC services utilization. We could not address the confounding effects of other potential factors that may act as a barrier for achieving recommended level of ANC services. Some of these potential factors include cost of care, availability and accessibility of health facilities, equity in health service delivery, gestational age or maturity of births, timing of ANC visits, and knowledge and attitudes towards modern health care services.

## Conclusion

This study documents unsatisfactory level of compliance with the WHO recommended minimum number of ANC visits and the core contents of the ANC visits during pregnancy in Bangladesh. Despite massive emphasis given to ANC under safe motherhood policies and programme by the government and non-government organization of Bangladesh, this study findings highlight that Bangladesh is still very far from achieving universal coverage of recommended ANC. However, utilization of health services is a complex phenomenon. It is related to the organization of the health care service delivery system, availability, quality, costs, health beliefs and comprehensiveness of services as well as socio-economic and demographic factors. Being a resource poor country, the maternal health care programme of the country still facing many challenges including, accessibility, lack of equity, lack of public health facilities, scarcity of skilled workforce and inadequate financial resource allocation [57]. More studies, both qualitative and quantitative, need to be under taken to identify the barriers of utilization of ANC services in Bangladesh and develop an effective ANC programme. This study identified many socio-economic and demographic factors that are related to the frequency of ANC visits and receiving of core elements of ANC services in Bangladesh. High socio-economic status, low parity, living in urban areas and certain administrative regions, planned pregnancies, having media exposure, visiting skilled providers for ANC services and visit to public or NGO health facilities are associated with frequent ANC visits and receiving higher number of items of ANC contents. There is a significant positive association between frequency of ANC visits and the utilization of higher number of items of ANC contents. Visiting skilled providers for ANC services and visit to public or NGO health facilities are also associated with frequent ANC visits and receiving higher number of items of ANC contents. The association between low socio-economic status and lower frequent ANC visits as well as receiving lower number of items of ANC contents suggests that poverty is an impediment to receiving appropriate antenatal care. Low performance of optimal use of ANC services among the mothers from rural areas and certain administrative region may also be related to their low socio-economic status and accessibility. The findings of lowerfrequency of ANC visits and lower number of items of ANC contents received in Sylhet, Rajshai and Barisal division underscore the requirement to develop policies specific to these regions. It is important to find out why these regions have lower prevalence of adequate care and develop specific solutions for these regions. Our findings suggest that, in the short term, less educated, socioeconomically disadvantaged, high parity mothers and mothers with unplanned pregnancy should be targeted to improve outcomes. However, for longer term improvement, there needs to be a focus on improving the education of females and access to quality ANC services. Promoting female education would be very effective in enhancing health care behaviour of women. Strengthening mass-media campaign on the benefits of adequate number of ANC visits and adherence to the recommended contents of ANC would further enhance the receiving of adequate maternal care. Our analysis indicates significant positive association between frequency of ANC visits received and the utilization of higher number of items of ANC services. The current practice of ANC program in Bangladesh is still based on WHO’s old guidelines of at least 4 ANC visits. For further improvement of maternal and child health, the ANC program in Bangladesh need to be redesigned in the light of updated WHO guidelines focusing on at least eight ANC visits and adequate level of ANC content. Having adequate level of ANC visits and ANC content may contribute to early detection and timely management of risk for adverse pregnancy outcome.
